# Neurochemistry Predicts Convergence of Written and Spoken Language: A Proton Magnetic Resonance Spectroscopy Study of Cross-Modal Language Integration

**DOI:** 10.3389/fpsyg.2018.01507

**Published:** 2018-09-04

**Authors:** Stephanie N. Del Tufo, Stephen J. Frost, Fumiko Hoeft, Laurie E. Cutting, Peter J. Molfese, Graeme F. Mason, Douglas L. Rothman, Robert K. Fulbright, Kenneth R. Pugh

**Affiliations:** ^1^Department of Special Education, Peabody College, Vanderbilt University, Nashville, TN, United States; ^2^Vanderbilt Brain Institute, Vanderbilt University School of Medicine, Nashville, TN, United States; ^3^Haskins Laboratories, New Haven, CT, United States; ^4^Department of Psychiatry, University of California, San Francisco, San Francisco, CA, United States; ^5^Peabody College of Education and Human Development, Vanderbilt University, Nashville, TN, United States; ^6^Vanderbilt Kennedy Center, Vanderbilt University, Nashville, TN, United States; ^7^Section on Functional Imaging Methods, Laboratory of Brain and Cognition, Department of Health and Human Services, National Institutes of Mental Health, National Institutes of Health, Bethesda, MD, United States; ^8^Department of Radiology and Biomedical Imaging, Yale University School of Medicine, New Haven, CT, United States; ^9^Department of Psychiatry, Yale University School of Medicine, New Haven, CT, United States; ^10^Department of Biomedical Engineering, Yale University School of Medicine, New Haven, CT, United States; ^11^Department of Psychological Sciences, University of Connecticut, Storrs, CT, United States

**Keywords:** magnetic resonance spectroscopy (MRS), reading, multisensory, cross-modal, reading disability (RD), developmental dyslexia

## Abstract

Recent studies have provided evidence of associations between neurochemistry and reading (dis)ability (Pugh et al., 2014). Based on a long history of studies indicating that fluent reading entails the automatic convergence of the written and spoken forms of language and our recently proposed Neural Noise Hypothesis (Hancock et al., 2017), we hypothesized that individual differences in cross-modal integration would mediate, at least partially, the relationship between neurochemical concentrations and reading. Cross-modal integration was measured in 231 children using a two-alternative forced choice cross-modal matching task with three language conditions (letters, words, and pseudowords) and two levels of difficulty within each language condition. Neurometabolite concentrations of Choline (Cho), Glutamate (Glu), gamma-Aminobutyric (GABA), and N- acetyl-aspartate (NAA) were then measured in a subset of this sample (*n* = 70) with Magnetic Resonance Spectroscopy (MRS). A structural equation mediation model revealed that the effect of cross-modal word matching mediated the relationship between increased Glu (which has been proposed to be an index of neural noise) and poorer reading ability. In addition, the effect of cross-modal word matching fully mediated a relationship between increased Cho and poorer reading ability. Multilevel mixed effects models confirmed that lower Cho predicted faster cross-modal matching reaction time, specifically in the hard word condition. These Cho findings are consistent with previous work in both adults and children showing a negative association between Cho and reading ability. We also found two novel neurochemical relationships. Specifically, lower GABA and higher NAA predicted faster cross-modal matching reaction times. We interpret these results within a biochemical framework in which the ability of neurochemistry to predict reading ability may at least partially be explained by cross-modal integration.

## Introduction

Most children in the United States education system begin the process of learning to read in kindergarten, a process that will continue formally in the classroom until they are 10 or 11 years old. Whereas learning to read requires explicit instruction, the ability to perceive and produce native language typically begins without instruction. Thus, children begin kindergarten with knowledge of their native speech sounds. Despite acquisition differences in listening and reading, it is well established that intact speech perception and production facilitates learning to read (Mattingly, 1971; Liberman, 1973). In fact, fluent reading requires learning the correspondence between letters and speech sounds (Marsh et al., 1981; Frith, 1985). Moreover, associations between auditory and visual letter learning jointly influence each other (Perfetti, 1987). Thus, a central role for learning letter-speech sound associations is highlighted in models of reading development (e.g., Ehri and Wilce, 1985; Share and Stanovich, 1995).

### Importance of cross-modal integration for reading

Information from different sensory modalities (e.g., visual and auditory inputs) must be integrated, assimilated, and organized as intersensory information. Early studies found that auditory-visual integration improves with age and, particularly relevant here, is correlated with reading skills (Birch and Belmont, 1965; see Kavale, 1980, 1982 for meta-analyses). Across 31 studies, Kavale (1980) reported a correlation between audio-visual integration and reading ability (*r* = 0.329, range: 0.025–0.617). In particular, one type of audio-visual integration, that of spoken and written language, has close ties to reading ability. Spoken-written language integration is often considered a separate, or special, type of intersensory “audio-visual” integration (see van Atteveldt et al., 2007; Froyen et al., 2010). This integration of spoken and written language has been shown at the level of the word (Frost et al., 1988), syllable (Massaro et al., 1988), and letter (see Blomert and Froyen, 2010 for review). Furthermore, letter-speech sound integration is often considered an early indicator of developmental reading outcome (see Blomert, 2011 for review).

Across a spectrum of reading ability, the poorest readers are those with a profound reading disability (RD; often referred to as developmental dyslexia) (see Gabrieli, 2009 for brief review). As previously mentioned, learning to read requires that unfamiliar visual symbols (i.e., letters) be associated with familiar auditory sounds (i.e., speech sounds). Thus, information must be integrated both within (i.e., unimodal or intramodal) and between (i.e., bimodal or intermodal) the auditory and visual sensory modalities. RD is historically characterized by a unimodal impairment, a deficit in phonological awareness: the use, manipulation, and processing of speech sounds (Bradley and Bryant, 1978; Liberman et al., 1989). However, Birch (1962) posited early on that reading impairment could be the result of a bimodal impairment. Specifically, the inability to integrate intersensory information could be indicative of reading impairment. In support of this idea, some studies have found that individuals with RD do struggle with auditory-visual integration (e.g., Birch and Belmont, 1965; Snowling, 1980; Siegel and Faux, 1989). Others have argued that an impairment in phonological awareness leads to an impairment in auditory-to-visual integration (e.g., Frith, 1985). In other words, an individual with a RD cannot adequately learn to perceive speech sounds, making it difficult to establish robust mappings between speech sounds and letter forms. However, children with a RD have shown unimpaired unimodal perception, in the form of auditory-auditory matching (Snowling, 1980; Siegel and Faux, 1989), visual-visual matching (Maurer et al., 2010), and have demonstrated typical letter mastery (Blomert and Willems, 2010). This indicates that unimodal perception, whether auditory or visual, does not encompass the difficulty underlying cross-modal integration. Therefore, cross-modal integration provides unique insight into both unimpaired and impaired reading development.

The development of cross-modal integration begins early. In children with typical reading abilities, electrophysiological responses to printed orthography are seen as early as first grade, when children are typically 6–7 years old (Maurer et al., 2006); this has been suggested to be the beginning of automation of the reading system (Chein and Schneider, 2005). Letter-speech sound associations are quickly learned (Ziegler and Goswami, 2005), and the neural responses accompanying these associations are adult-like by second grade (Maurer et al., 2006). Although automatization of this integrated process extends further into development (Booth et al., 2001; Froyen et al., 2008), to capture the early stages in the process of developing cross-modal neural responses, cross-modal interaction would need to be studied during early elementary school. While typically developing children quickly learn the relationship between processing auditory and visual letters, for poorer readers and children with RD this trajectory is less straightforward. When individuals with a RD were asked to complete cross-modal tasks, it was discovered that they had irregular letter-speech sound integration at the beginning of reading development, which remained irregular into adulthood (Blau et al., 2009, 2010). Perhaps most intriguing, reading disabled children's cross-modal integration was found to decrease over the course of reading instruction suggestive of an entirely different cross-modal development trajectory in typical readers compared to those with reading impairments (see Blomert, 2011 for review).

### Links between neurochemistry and reading

There is a multitude of evidence suggesting a biological basis of reading ability and disability, yet the exact biological mechanisms remain unknown. One particular method that has promise for understanding biological mechanisms is proton (1H) Magnetic Resonance Spectroscopy (MRS), a non-invasive technique used to measure biochemical resonance levels and determine neurometabolite concentrations *in vivo*. Across developmental disorders, neurometabolites concentrations have been found to vary compared to their typically developing age-matched peers (Perlov et al., 2009; Baruth et al., 2013). Thus far, a limited number of studies have investigated the relationship between neurometabolite concentrations and reading (see Del Tufo and Pugh, 2012 for review). In adults, levels of Choline (Cho) were higher for those with poorer phonological ability (Bruno et al., 2013), and higher in individuals with RD relative to their typical developing peers (Rae et al., 1998; Laycock et al., 2008). See Table 1: H1-MRS Findings in Reading and Reading Disability. In an initial study from our group, Pugh and colleagues explored this relationship in emergent readers, establishing that higher levels of Cho measured in a midline occipital region was negatively correlated with children's reading ability. Moreover, Pugh et al. (2014) found that reading skill is negatively correlated with glutamate (Glu), a neurometabolite that is involved in a large number of neuronal metabolic pathways and can be used to explain system excitability.

**Table 1:** H1-MRS findings in reading and reading disability.

**Paper**	**Participants**	**NM**	**SVS**	**Primary findings**
Pugh et al., 2014	Children across a spectrum of reading ability at visit 1 (*n* = 75, mean age 7.68 years).	Cho Cr NAA GABA Glu	Midline occipital cortex (includes: lingual gyrus, calcarine sulcus and cuneus)	Increased Cho:Cr and Glu:Cr were associated with decreased reading ability.
	Children across a spectrum of reading ability at follow up^§^ (*n* = 45, mean age 10.1 years).			Increased Glu:Cr was associated with decreased reading ability.
	RD vs. TD Children at Visit 1 (*n* = 47, 10 RD).			RD children had increased Cho:Cr and Glu:Cr.
	Pediatric Readers* across a spectrum of reading ability. (*n* = 85, 5–18 years).	Cho Cr NAA	Midline occipital region	Increased Cho:Cre was associated with decreased reading ability.
Nakai and Okanoya, 2016	Adults (*n* = 28, 18–22 years).	GABA Cr	L. IFGR. IFG	Negative correlation between verbal category fluency** and GABA:Cr in the L. IFG.
Bruno et al., 2013	Adults across a spectrum of reading ability (*n* = 31, 18–30 years, 10 RD).	Cho Cr NAA	L. Angular Gyrus	Increased Cho:Cr was associated with increased phonological ability.
Lebel et al., 2016	Children (*n* = 56, 3.0–4.7 years).	Glutamate Glutamine Cr Cho Inositol NAA	Anterior Cingulate Gyrus	Increased Glu, Cr, and Inositol were associated with increased phonological processing (NEPSY-II).
	Children (*n* = 45, 3.2–5.4 years)		L. Angular Gyrus	Increased Cho and Glutamine were associated with decreased speeding naming (NEPSY-II).
Rae et al., 1998	RD vs. TD adults (*n* = 29, 21–40 years, 14 RD).	Cho Cr NAA	L. Temporoparietal Lobe R. Temporoparietal Lobe L. CerebellumR. Cerebellum	Decreased Cho:NAA in RD in the L. temporoparietal lobe.Decreased Cho:NAA and Cr:NAA in RD in the R. cerebellum.
Laycock et al., 2008	RD vs. TD adults (*n* = 12, mean age 21.1 years, 6 RD).	Cho Cr NAA	R. CerebellumL. Cerebellum	RD had lower NAA:Cho in the R. cerebellum and higher Cho:Cr in the L. cerebellum.
Richards et al., 1999	RD vs. TD children (*n* = 13, 6 RD).	Lactate NAA	Sylvian fissure	Increased Lactate:NAA in RD in the sylvian fissure. However, this relationship was found only during a rhyming task, not during the lexical decision task or at rest.

*NM, Neurometabolite; SVS, small voxel spectroscopy; RD, Reading Disability; TD, Typically Developing; Cho, Choline; Cr, Creatine; NAA, N-Acetylaspartate; GABA, gamma-Aminobutyric acid; Glu, Glutamate; L, left; R, Right*.

* *Pediatric readers from the NIH MRI Study of Normal Brain Development (http://pediatricmri.nih.gov, release 5)*.

§ *Follow up assessments took place twenty-4 months post-initial assessment*.

** *Category Fluency Task: Native Japanese Speakers had 1 min to write down as many Japanese nouns as possible belonging to each category: animal, fruit, and vehicle*.

Hancock et al. (2017) have recently proposed a “Neural Noise Hypothesis of Developmental Dyslexia.” The precis of this hypothesis is that increased neural excitability, which leads to neural noise in cortical networks, is a key contributor to RD. In their hypothesis, “neural noise” refers to random variability in neuronal firing. Although the specifics of the underlying biochemical mechanism are not yet fully established, they offer examples of genetic pathways from two highly replicated dyslexia candidate genes (*DCDC2* and *KIAA0319*), both known to affect neural noise. *DCDC2* mutations increase neural noise through a direct effect on glutamatergic signaling and hyperexcitability (Meng et al., 2005; as evidenced by Che et al., 2014, 2016). *KIAA0319* mutations disrupt neural migration and the formation of local excitatory-inhibitory circuits (Paracchini et al., 2006; Peschansky et al., 2010; Huang and Hsueh, 2015). Hancock et al. (2017) posited that increased neural noise leads to disruptions in neural synchronization and precise neural spike timing. This would in turn lead to impairment in phonological awareness and particularly relevant here, multisensory integration. The hypothesis further predicts that the impairment in multisensory integration may arise from disruptions in visual or auditory sensory areas. However, beyond the dual points of susceptibility (i.e., visual and auditory), multimodal integration and coordination of processing across cortical regions are particularly sensitive to the loss of spike timing precision (Senkowski et al., 2007a,b). In summary, increased neural noise is hypothesized to lead to imprecise orchestration of multisensory information, resulting in disrupted multisensory integration.

### The current study

Our overarching goal was to determine the relationship between neurometabolite concentrations and cross-modal integration in emergent readers. Based on the “Neural Noise Hypothesis of Developmental Dyslexia” (Hancock et al., 2017), we hypothesized that diminished multisensory integration would correspond to increased Glu levels–a proximal measure of increased glutamatergic signaling and hyperexcitability. Our full sample of emergent readers (*n* = 231) completed a behavioral cross-modal matching task. After validating our behavioral cross-modal task we then used a subsample of those participants (*n* = 70; those that also contributed MRS data) to determine if emergent readers' neurochemistry (Glu, GABA, Cho, NAA) predicted differences in cross-modal matching. Next, we used structural equation modeling (SEM) to determine if the relationship between emergent readers' neurochemistry and reading ability was mediated by their performance on the cross-modal integration task. Finally, follow-up analyses of our initial SEM mediation model investigated if children's reading ability was driven by specific cross-modal stimuli integration and predicted by specific neurometabolite concentrations.

## Materials and methods

### Participants

Researchers obtained parental informed consent and child assent in compliance with Yale University's Human Research Protection Program. Parental report indicated that all children were native speakers of American English with normal or corrected-to-normal vision, normal hearing, and no history of neurological or mood disorders. All children had a performance intelligence quotient (PIQ) within normal limits. Children were recruited through the Yale Reading Center in order to recruit across a diverse range of reading ability from good-to-impaired. See Table 2 for participant demographics and descriptive statistics.

**Table 2:** Demographic and descriptive statistics.

**Characteristics**	**Full sample (*n* = 231)**	**Subsample (*n* = 70)**
Sex	132 male;99 female	44 male;26 female
Age	8.14 (1.42)	7.70 (0.71)
**STANDARDIZED ASSESSMENT SCORES**		
WASI: Performance IQ	110.77 (15.54)	108.58 (16.68)
WASI: Full Scale IQ	109.15 (16.89)	109.62 (17.62)
TOWRE: Sight Word Efficiency	50.48 (20.13)	47.23 (19.32)
TOWRE: Phonemic Decoding Efficiency	23.80 (14.33)	21.59 (13.32)
WJ-III: Letter-Word Identification	44.66 (13.02)	43.07 (11.37)
WJ-III: Word Attack	17.59 (7.52)	16.84 (6.77)

*Sample mean and (standard deviation) are reported. Performance IQ and Full Scale IQ are from the Wechsler Abbreviated Scale of Intelligence (WASI: Wechsler, 1999). Single word and pseudoword reading ability raw scores are reported for both timed (TOWRE: Torgesen et al., 1999) and untimed (Woodcock-Johnson Test of Achievement III; Woodcock et al., 2001) measures*.

Of the 231 participants [132 male, mean *(M)* age = 8.14 years, standard deviation *(SD)* = 1.42] who performed the cross-modal matching task, seven participants failed to respond during the cross-modal matching task. An additional two participants failed to complete the word condition. Three additional participants failed to complete the pseudoword condition. The remaining participants all scored above chance on the cross-modal matching task (chance = 50% accuracy). Thus, our full sample analysis of the behavioral cross-modal matching task included 224 children for the letter stimulus condition, 222 children for the word stimulus condition, and 221 children for the pseudoword stimulus condition. Of the full sample of cross-modal matching task participants, a subset of those reported in Pugh et al. (2014) also contributed MRS data. Of those 70 participants [44 male, *(M)* age = 7.70 years, *SD* = 0.71], one participant failed to respond during the cross-modal matching task. Of those 69 children, one child scored below chance in the word stimulus condition, and two children scored below chance on the pseudoword stimulus condition. Therefore, our subsample analyses included 69 children for the letter stimulus condition, 68 children for the word stimulus condition, and 67 children for the pseudoword stimulus condition. Of those 69 subjects, three contributed partial metabolite spectra [Cho (*n* = 67), Glu (*n* = 66), GABA (*n* = 69), and NAA (*n* = 66)] due to poor spectral quality (see MRS methods below for spectral quality details).

### Cross-modal integration task

**Figure 1:**
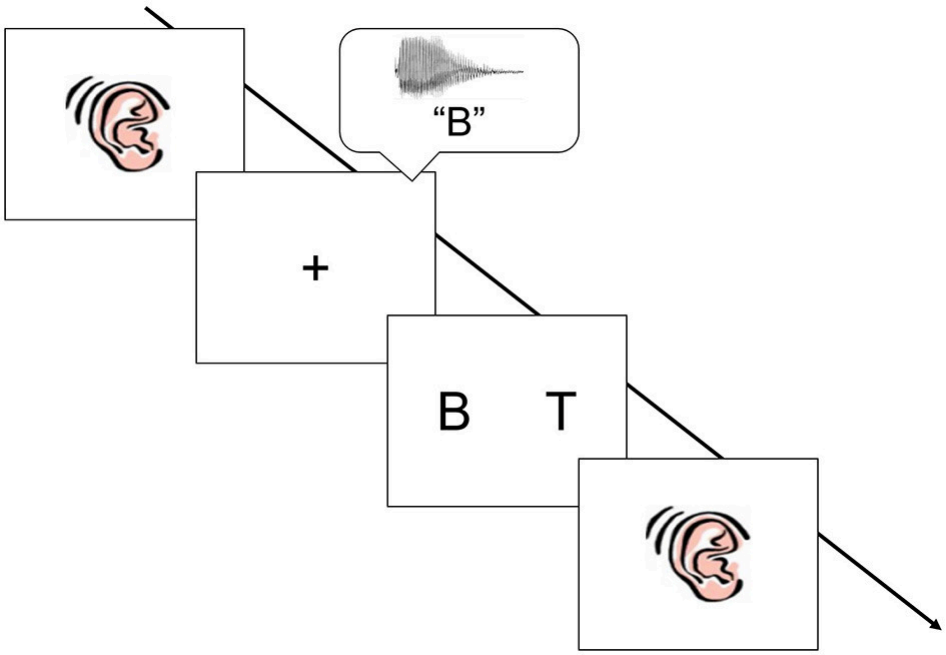
Schematic of the cross modal matching task. A picture of an ear appeared in the center of screen. An auditory stimulus was heard (spoken letter name, word, or pseudoword), followed by two visual stimuli. Children were asked to make a choice judgment: was the visual stimulus on the right or the visual stimulus on the left a match to the auditory stimulus. The stimuli were presented in three sequential blocks: letters, words, and then pseudowords.

A two-alternative forced choice task was designed to assess auditory-visual cross-modal matching (see Figure 1; Shaywitz et al., 2004). During the experiment a picture of an ear appeared in the center of the screen for 1,500 milliseconds (ms), followed by an auditory spoken letter name, word, or pseudoword presented binaurally through headphones (e.g., “B”). The auditory stimulus was then followed 1,000 ms later by two visual target stimuli (e.g., “B” and “T”). The visual stimuli were offset by 10° of center to the right and left, respectively. The two visual stimuli remained on the screen until either the child responded, or a period of 4,000 ms had passed. The inter-stimulus interval (ISI) was 1,000 ms and immediately followed either a response or the 4,000 ms time lapse. The experiment was divided into three blocks, with each stimulus condition (letters, words, and pseudowords) comprising an experimental block.

Children were instructed to respond as quickly as possible by pressing the button that corresponded to the position (right or left) of the visual stimulus that matched the spoken letter name. To avoid fatigue effects, children received a 1-to-2-min break between each experiment block. In the first block children heard and saw letters, in the second they heard and saw words, and in the third they heard and saw pseudowords. To ensure that children understood the task directions, children viewed an instructional flipbook and competed practice items immediately prior to the cross-modal matching task. The flipbook and practice items included trial examples for each of the three conditions and children received feedback as to whether or not they were correct.

#### Stimuli

The letter block included all 26 English letters as stimuli. The word and pseudoword blocks included consonant-vowel-consonant (CVC) stimuli (see Appendix A in Supplementary Material for stimuli). Two conditions were included in each experiment block. The first condition was degree of difficulty. All three blocks contained easier (14 stimulus pairs) and more difficult (14 stimulus pairs) visual stimulus pairs to match. Easy stimulus pairs had no overlap in orthography or phonology (e.g., BAM, ROG). Hard stimulus pairs overlapped in either phonology (letters) or orthography and phonology (words and pseudowords; e.g., BAL, BAF). The second condition was repetition. In each experiment block, stimuli were fully randomized and presented once (first stimulus presentation) and then randomized and presented for the second time (second stimulus presentation).

#### Counterbalancing

Two experiment versions (A and B) were created, for counterbalancing. Matching targets that were a “hard” stimuli pair in one experimental version instead formed an “easy” stimuli pair in the other experiment version. For example, BAL, BAF (a hard pseudoword pair) in one experimental version would become BAL, MOT (an easy pseudoword pair) in the other experimental version. Likewise, matching targets that were an “easy” stimuli pair in one experimental version instead formed a “hard” stimuli pair in the other experiment version. For example, BAL, MOT (an easy pseudoword pair) in one experimental version would become BAL, BAF (a hard pseudoword pair) in the other experimental version. Additionally, the limited set of letter stimuli that are confusable (or not) made counterbalancing impossible for the letter condition. Thus, counterbalancing only applied to the word and pseudoword conditions.

### Magnetic resonance methods

A 4T Bruker Avance Magnetic Resonance system was used to acquire MR spectroscopy. All participants watched a commercially available movie, without sound, to encourage stillness and relaxation. A spin-echo J-editing acquisition sequence (Rothman et al., 1993) was used to measure the metabolite basis signal for all neurometabolites: edited GABA and non-edited Cho, Cr, NAA, and Glu. An H-tuned surface coil (7-cm) was used to increase sensitivity. To position the voxel, gradient echo scout images were acquired (slice thickness 1.5 mm with no gap and a field of view 200 mm, divided into 128 × 128 pixels). The volume of interest was a 3 × 3 × 1.5 cm voxel placed at the midline of the occipital cortex, including the lingual gyrus, calcarine sulcus, and cuneus (see Pugh et al., 2014 for image of voxel placement and spectra). Eckert et al. (2008) and others have shown that this central occipital region correlates with activation in left Heschl's gyrus (Zangenehpour and Zatorre, 2010; Murray et al., 2016). Results that are consistent with anatomical evidence from non-human primates (see van Wassenhove et al., 2012 for review). The water signal was used to calibrate the pulse power for MR spectroscopy.

#### Quantitative T1 sequences

Rapid inversion-recovery sampling was used to obtain quantitative T1 (Mason and Rothman, 2002), which are optimized for statistical sensitivity (Mason et al., 1997). A B1 map was acquired to correct for surface coil inhomogeneities. Quantitative T1 images were converted to graded segmented images: percentage gray matter, white matter, and cerebrospinal fluid (Mason and Rothman, 2002). Based on the known dimensions and positions of the MRS voxel (see MRS Sequences below), the composition of the MRS voxel was determined from the segmented images as percentage gray matter, white matter, and cerebrospinal fluid (Mason and Rothman, 2002).

#### MRS sequences

Shimming was performed using FASTERMAP (Shen et al., 1997). The water signal was suppressed via six applications of chemical shift selected sequence (CHESS) using a 1,000 Hz offset swept amplitude pulse. Volume excitation employed a slice selective Shinnar-Le Roux pulse, followed by a 180° slice selective pulse. The 3D volume selection was obtained using outer volume suppression and image selected volume spectroscopy. Volume suppression outside of the voxel used an adiabatic full passage pulse in x, y, and z directions. A J-editing sequence (Rothman et al., 1993) was used to acquire the GABA resonance. The subspectra with (and without) editing inversion of the GABA C3 resonance were acquired: 1,024 data points in 410 ms, a 3 s repetition time, and a 68 ms echo time. To eliminate contamination by macromolecules, the DANTE editing pulse, which was placed symmetrically about the refocusing pulse, was applied at 1.89 and 1.31 ppm on alternative 8-scan blocks (Henry et al., 2001). The total acquisition period was 22 min.

#### Neurometabolite analyses

Linear combination spectral fitting was applied to the subspectrum obtained with the DANTE pulse applied at 1.31 ppm to determine the area of the resonances of Cho, Glu, NAA, and Cr. The unedited subspectrum was fitted using a basis set of metabolite spectra. The fitted metabolites included aspartate, glutamate, glutamine, N-acetyl-aspartate, N-acetyl-aspartyl-glutamate (NAAG), creatine, phosphocreatine, myoinositol, choline, phosphorylcholine, glycerophosphorylcholine, and scylloinositol. The J-editing acquisition sequence was employed to measure the metabolite basis signals, with the exception of NAA and phosphocreatine, which were simulated. Reported NAA was the combination of N-acetyl-aspartate and NAAG, reported Cr was the combination of creatine and phosphocreatine, and reported Cho was the combination of choline, phosphorylcholine, and glycerophosphorylcholine. Three subjects contributed partial metabolite spectra due to poor spectral quality. Following spectra fitting, a Monte-Carlo analysis was used to assess uncertainties of individual measurements. In the Monte-Carlo analysis the least-squares spectral fits were treated with random Gaussian noise whose standard deviation was equal to that of the raw data and refitted using 20 repetitions to estimate the SDs of the uncertainty for each metabolite measure. No data exceeded the criterion for exclusion—standard deviation greater than three times the average standard deviation for the full set of studies (Valentine et al., 2011).

GABA in the edited subtraction spectrum was analyzed with in house software written in MATLAB (www.mathworks.com). Each free induction decay (FID) was phased-locked using the water FID and frequency aligned using resonance from NAA, Cr, and Cho. Each pair of subspectra (27/experiment) was subtracted to obtain FID of the edited GABA signal, and then apodized. For quality control, sub-spectra pairs were excluded if their difference in GABA spectra showed residual intensity from either Cho or creatine in the subtraction spectrum (absorptive and dispersive), which also minimizes the effects of motion. The remaining spectra were then combined. The area of the GABA resonance at 3 ppm was determined using automated manual integration following automated baseline correction. GABA was determined in each subject. Two methods were used to evaluate macromolecular contamination: metabolite nulling (Behar et al., 1994; Rothman, 1994; Shen et al., 2004) and frequency switching symmetrically about the coupled macromolecular resonance (Henry et al., 2001). Neither method showed evidence of macromolecular contamination of the resonance.

The area of Cr was used as an internal reference, controlling for potential drifts in the spectra during acquisition (Rothman et al., 1993). Glu, Cho, NAA, and GABA are reported as a ratio of their metabolite resonance area relative to the internal Cr reference, as recommended by Rothman et al. (1993).

### Statistical analyses

Multilevel mixed effect models were employed using the maximum likelihood estimation (R: https://www.r-project.org, lme4 package: Bates et al., 2015). In all models, subjects were specified as the random intercept. This also controlled for associated intraclass correlation (Pinheiro and Bates, 2000). We employed forward-fitting model procedures to determine the model of best fit using likelihood ratio tests. Additionally, structural equation models of mediation were fitted using the R lavaan package (Rosseel, 2012), which uses a maximum likelihood estimation. Standard errors were calculated using bootstrapping procedures.

#### Multilevel mixed effect modeling task effects

In our initial mixed effect model analysis, we validated our cross-modal matching task. In this model all effects and their interactions were tested for improvement in model fit. Following a natural log transformation, there was no evidence of cross modal reaction time (CM-RT), the dependent variable, violating normality across stimulus repetitions [Full sample (*n* = 224): *Bartlett's test K*${}_{\left(1\right)}^{2}$ = 1.89, *p* = 0.17 and Subset sample (*n* = 69): *Bartlett's test K*${}_{\left(1\right)}^{2}$ = 0.0013, *p* = 0.97]. Due to differences in how the easier and more difficulty stimulus pairs were created within the three stimuli conditions (i.e., letters, words, and pseudowords), we considered the degree of difficulty factor to be nested within each condition. Repetition was included as a crossed factor.

#### Multilevel mixed effects modeling task effects predicted by NT

After validating the effect of our cross-modal matching task, we used a second mixed effect model to investigate if emergent readers' neurochemistry (Glu, GABA, Cho, NAA) predicted differences in cross-modal matching. Prior to inclusion in the models, fixed magnitude correlations were run on the z-scored neurometabolite concentrations of Glu, Cho, NAA, and GABA to determine if the magnitude of the overlap between correlations would require separate models to examine the respective effects of neurometabolite concentration on cross-modal matching. The neurometabolite concentrations did correlate (see Table 3: Neurochemical Concentration Correlation) but did not remove one another's unique variable contributions. Thus, neurometabolite concentrations were included as fixed effects in a single model.

**Table 3:** Correlations between neurochemical concentration.

**Neurochemicals**	**Cho**	**Glu**	**GABA**	
Cho				
Glu	*r* = 0.443**			
GABA	*r* = 0.151	*r* = 0.434**		
NAA	*r* = 0.230	*r* = 0.531**	*r* = 0.300*	

Spearman's pairwise correlations with p-values adjusted for multiple comparison (Holm's method). All neurometabolites are referenced to a Creatine (Cr) baseline. Significance:

* *p < 0.05*,

** *p < 0.01*,

*** *p < 0.001*.

#### Structural equation modeling mediation

Mediation analyses using Structural Equation Modeling (SEM) determined whether the relationship between emergent readers' neurometabolite concentration (a latent variable) and reading ability, which has been previously reported in Pugh et al. (2014), was mediated by CM-RT. Mediation models tested if the relationship between neurometabolite concentrations and reading ability was mediated by cross-modal matching, for each cross-modal stimulus condition. Mediation assumes that the mediating variable (CM-RT) causes the outcome variable (reading ability). The initial assumptions of mediation were met (see Baron and Kenny, 1986); namely, (a) neurometabolite concentrations (independent variable: IV) were found to be significantly related to cross-modal integration (mediating variable: MV), and (b) neurometabolite concentrations (IV) were found to be significantly predictive of reading ability (dependent variable: DV). Our prior analysis led us to expect CM-RT (MV) would have individual variation; thus, a latent variable approach to mediation was used (see Hayes, 2009 for review).

## Results

### Cross-modal matching task effects

We examined CM-RT predicted by task effects (i.e., stimulus condition, stimulus repetition, and degree of difficulty) performed by the full sample of children who completed the cross-modal matching task. See Table 4 for CM-RT by stimulus condition. We remind our reader that this included 224 children for the letter stimulus condition, 222 children for the word stimulus condition, and 221 children for the pseudoword stimulus condition. Multilevel mixed effect models, with subject as a random intercept and stimulus condition as a random slope, were employed to examine individual differences in emergent readers cross-modal matching. CM-RT showed significant variance in intercepts across participants and significant variance in slope across stimulus conditions *X*${}_{\left(5\right)}^{2}$ = 1464.4, *p* < 0.001. Thus, in addition to subject as the random intercept, stimulus condition was included as the random slope. The best fitting model included the fixed effects: stimulus condition (letters, words, and pseudowords), degree of difficulty (easy and hard stimulus pairs) nested by stimulus condition, and the two-way interaction of stimulus condition by repetition (first and second stimulus presentation) *X*^2^ = 48.52, *df* = 16 *p* < 0.001, *marginal R*^2^ = 0.18, and *conditional R*^2^ = 0.89. No improvement in model fit was found for the inclusion of the counterbalanced experiment version (A and B) factor (*p* = 0.60) nor for the inclusion of the three-way interaction: stimulus condition by repetition by degree of difficulty (*p* = 0.49).

**Table 4:** Cross modal task reaction time by stimulus condition.

**Condition**	**Full sample (*****n*** = **224)**			**Subsample (*****n*** = **69)**		
	**Reaction time (ms)**			**Reaction time (ms)**		
	***n***	***M***	***SD***	***n***	***M***	***SD***
Letter	224	920.65	292.71	69	960.37	274.69
Word	223	1185.09	450.48	68	1213.54	408.81
Pseudoword	221	1309.93	453.28	67	1336.56	415.02

*Includes only those who scored above chance. Sample mean (M) and standard deviation (SD) are reported. Reaction Time was measured in milliseconds (ms)*.

**Figure 2:**
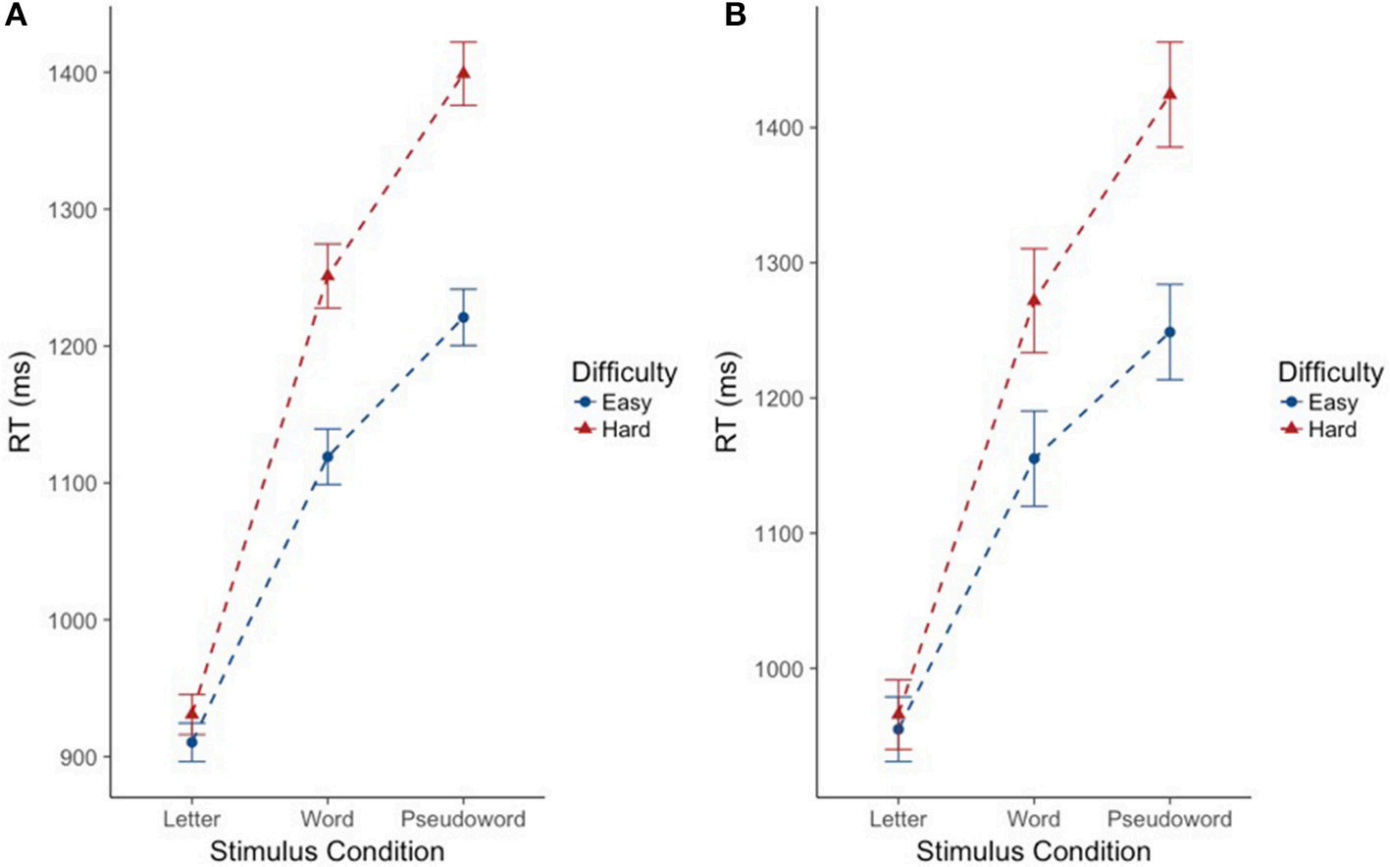
Effect of degree of difficulty on cross modal reaction time differs for words and pseudowords, but not letters. Reaction time is reported in milliseconds (ms) on the y-axis. Stimulus condition is reported on the x-axis. Difficulty is indicated by color and shape. The “hard” condition is in red triangles. The “easy” condition is in blue circles. Error bars reflect standard error of the mean (SEM). **(A)** Includes the full sample of participants (*n* = 224). **(B)** Includes the subsample of participants (*n* = 69).

**Figure 3:**
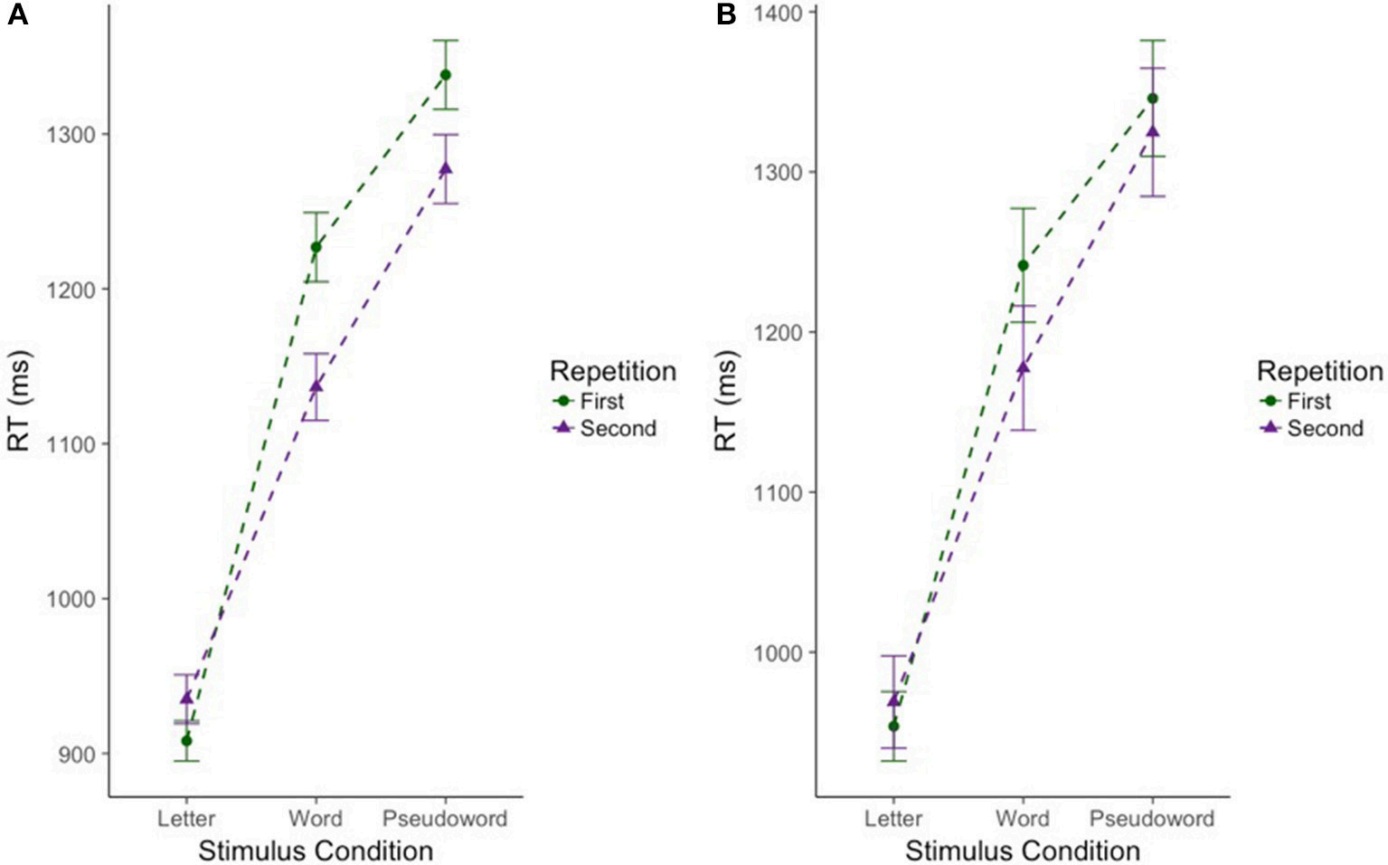
Effect of repetition on cross modal reaction time differs for words and pseudowords only in the full sample of participants. Reaction time is reported in milliseconds (ms) on the y-axis. Stimulus condition is reported on the x-axis. Repetition is indicated by color and shape. The first presentation of a stimulus is in green circles. The second presentation of a stimulus is in purple triangles. Error bars reflect standard error of the mean (SEM). **(A)** Includes the full sample of participants (*n* = 224). **(B)** Includes the subsample of participants (*n* = 69).

As expected, there was a significant effect of stimulus condition *F*_(2, 220.39)_ = 192.74, *p* < 0.001. Bonferroni *post-hoc* tests confirmed that CM-RT for the letter stimulus condition was faster than CM-RT for both the word stimulus condition [*b* = −0.24, *SE* = 0.017, *t*_(219.94)_ = 13.99, *p* < 0.001] and the pseudoword stimulus condition [*b* = −0.34, *SE* = 0.02, *t*_(218.95)_ = 19.49, *p* < 0.001]. Additionally, CM-RT for the word stimulus condition was faster than for the pseudoword stimulus condition [*b* = −0.11, *SE* = 0.011, *t*_(217.95)_ = 9.50, *p* < 0.001]. Therefore, as expected, CM-RT was fastest for the letter condition, followed by the word condition, and slowest for pseudowords. Nested within stimulus condition, there was a significant effect of degree of difficulty *F*_(3, 1823.14)_ = 119.99, *p* < 0.001. This was driven by faster cross-modal matching CM-RT on the easy stimuli compared to the hard stimuli in all three stimulus conditions: letter [*b* = −0.02, *SE* = 0.009, *t*_(1823.14)_ = 2.22, *p* < 0.05], word [*b* = −0.11, *SE* = 0.009, *t*_(1823.14)_ = 11.70, *p* < 0.001], and pseudoword [*b* = −0.14, *SE* = 0.009, *t*_(1823.14)_ = 14.77, *p* < 0.001] (Figure 2A). Thus, across each stimulus condition we see slower CM-RT for the hard stimuli. Repetition was not included as a fixed effect as there was no increase in model fit when repetition was included on its own (*p* = 0.21), but there was an interaction of stimulus condition by repetition *F*_(2, 1872.41)_ = 16.39, *p* < 0.001. The two-way stimulus condition by repetition interaction was driven by faster CM-RT in the letter condition for the first compared to the second letter stimulus presentation [*b* = 0.05, *SE* = 0.01, *t*_(1864.87)_ = 4.71, *p* < 0.001]. Conversely, faster CM-RT for the second compared to the first stimulus presentation drove the interaction in the word [*b* = −0.04, *SE* = 0.01, *t*_(1879.21)_ = 4.63, *p* < 0.001], and pseudoword conditions [*b* = −0.02, *SE* = 0.01, *t*_(1876.73)_ = 2.36, *p* < 0.05] (Figure 3A). Thus, we find that during the letter stimulus condition, CM-RT for the first stimulus repetition is faster than the CM-RT for the second stimulus repetition. Conversely, in the word and pseudoword stimulus conditions, CM-RT for the second stimulus repetition is faster than the CM-RT for the first stimulus repetition. The results of the interaction also explain the lack of effect of repetition on its own. The effect of repetition is reversed during letter stimulus condition as compared to the word and pseudoword stimulus conditions. This suggests that children are taking advantage of the effect of stimulus repetition only for the word and pseudoword stimulus conditions.

### Neurometabolite concentrations predict cross-modal matching

Next, we examined the effect of neurometabolite concentrations on CM-RT. This analysis included only the subsample—the subset of individuals who scored above chance on our cross-modal matching task and contributed MRS data: Cho (*n* = 67), Glu (*n* = 66), GABA (*n* = 69), and NAA (*n* = 66). This included 69 children for the letter stimulus condition, 68 children for the word stimulus condition, and 67 children for the pseudoword stimulus condition.

CM-RT showed significant variance in intercepts across participants and significant variance in slope across stimulus conditions *X*${}_{\left(5\right)}^{2}$ = 1349.45, *p* < 0.001. The best fitting model included the following fixed effects: stimulus condition (letters, words, and pseudowords), neurometabolite concentrations (GABA and NAA), degree of difficulty (easy and hard stimuli) nested by stimulus condition, the two-way interaction of stimulus condition by neurometabolite concentrations, and finally the three way interaction of Cho concentration by degree of difficulty nested by stimulus condition *X*^2^ = 80.07, *df* = 25, *p* < 0.001, *marginal R*^2^ = 0.30, and *conditional R*^2^ = 0.85. As in the previous (full sample) analysis, there was a significant effect of stimulus condition *F*_(2, 64.10)_ = 55.85, *p* < 0.001. Bonferroni *post-hoc* tests confirmed that CM-RT for the letter stimulus condition was faster than CM-RT for both the word stimulus condition [*b* = −0.21, *SE* = 0.027, *t*_(62.79)_ = 7.90, *p* < 0.001] and the pseudoword stimulus condition [*b* = −0.32, *SE* = 0.03, *t*_(59.61)_ = 10.35, *p* < 0.001]. There was also a significant effect of degree of difficulty nested within stimulus condition *F*_(3, 499.97)_ = 26.15, *p* < 0.001. This was driven by faster CM-RT on the easy stimuli compared to the hard stimuli in the word [*b* = 0.099, *SE* = 0.02, *t*_(499.97)_ = 5.12, *p* < 0.001] and pseudoword [*b* = 0.14, *SE* = 0.02, *t*_(499.97)_ = 7.21, *p* < 0.001] stimulus conditions (Figure 2B). There was no effect of repetition by itself, nor was there an increase in model fit for the interaction of stimulus condition by repetition (Figure 3B).

**Figure 4:**
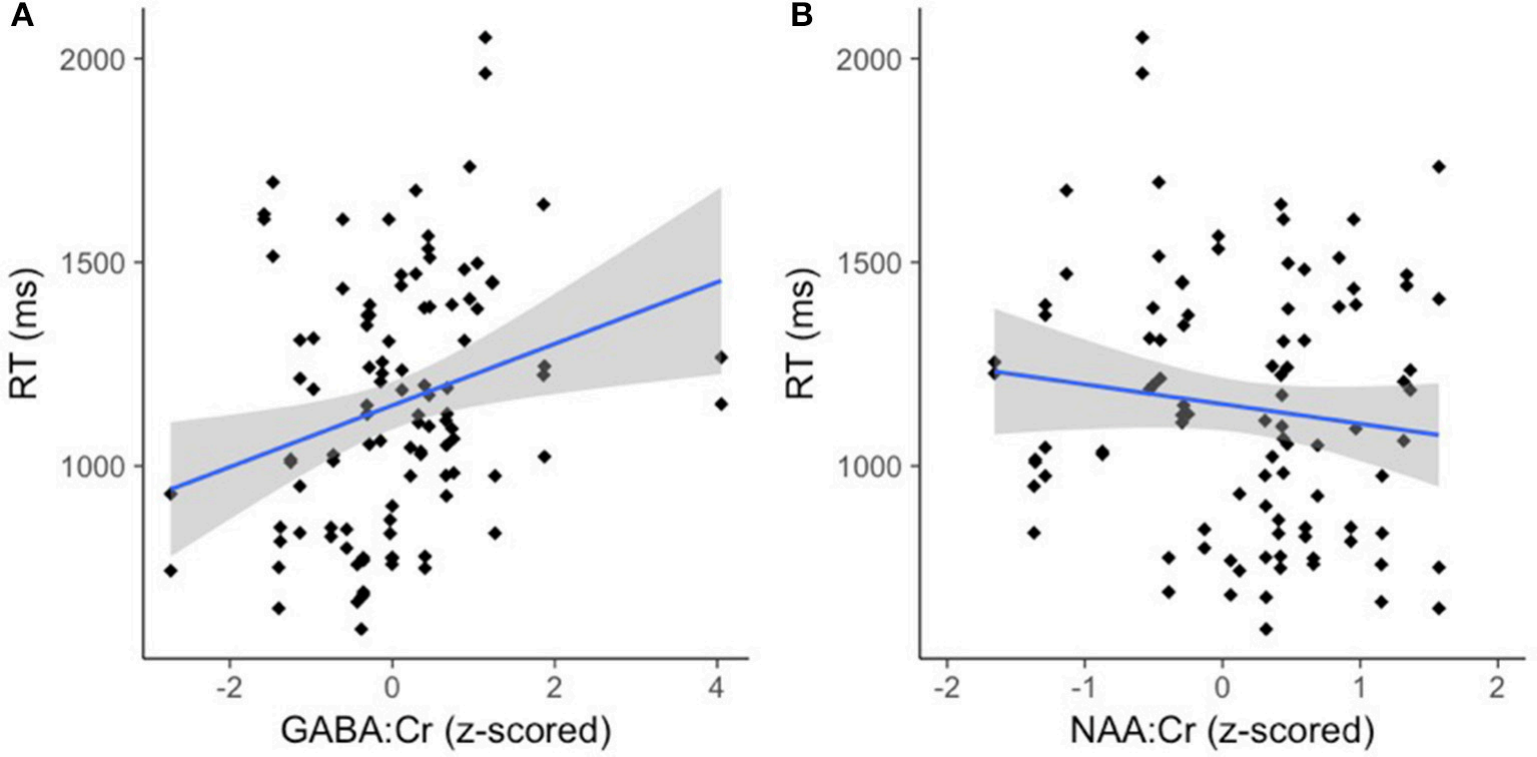
Lower GABA and higher NAA concentration predict faster cross-modal reaction time. Reaction time is reported in milliseconds (ms) on the y-axis. **(A)** GABA:Cr concentration is reported on the x-axis. **(B)** NAA:Cr concentration is reported on the x-axis. The gray area reflects the standard error of the mean (SEM).

**Figure 5:**
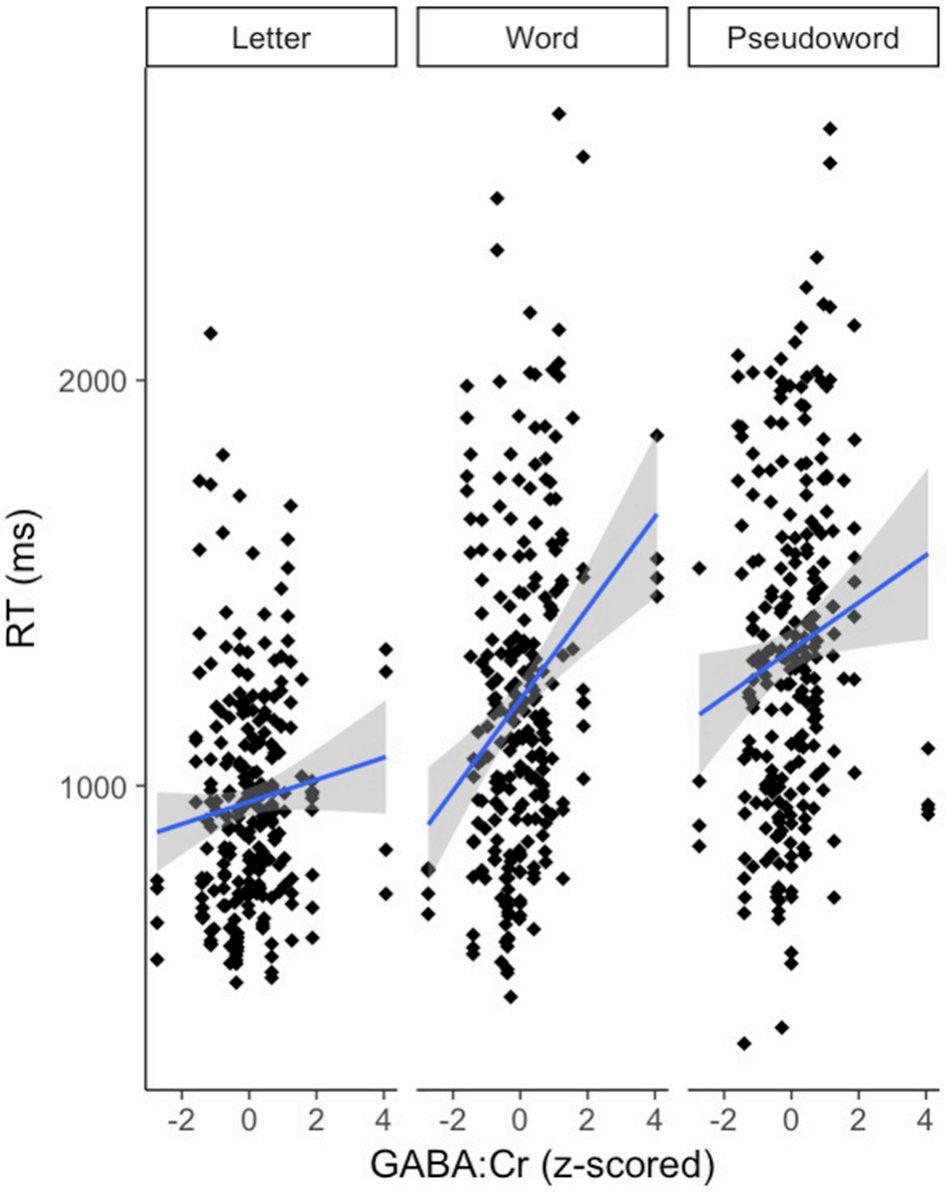
Effect of GABA on cross modal reaction time is driven by the word stimulus condition. Reaction time is reported in milliseconds (ms) on the y-axis. GABA:Cr concentration is reported on the x-axis. Stimulus condition is reported at the top of each panel. The gray area reflects the standard error of the mean (SEM).

**Figure 6:**
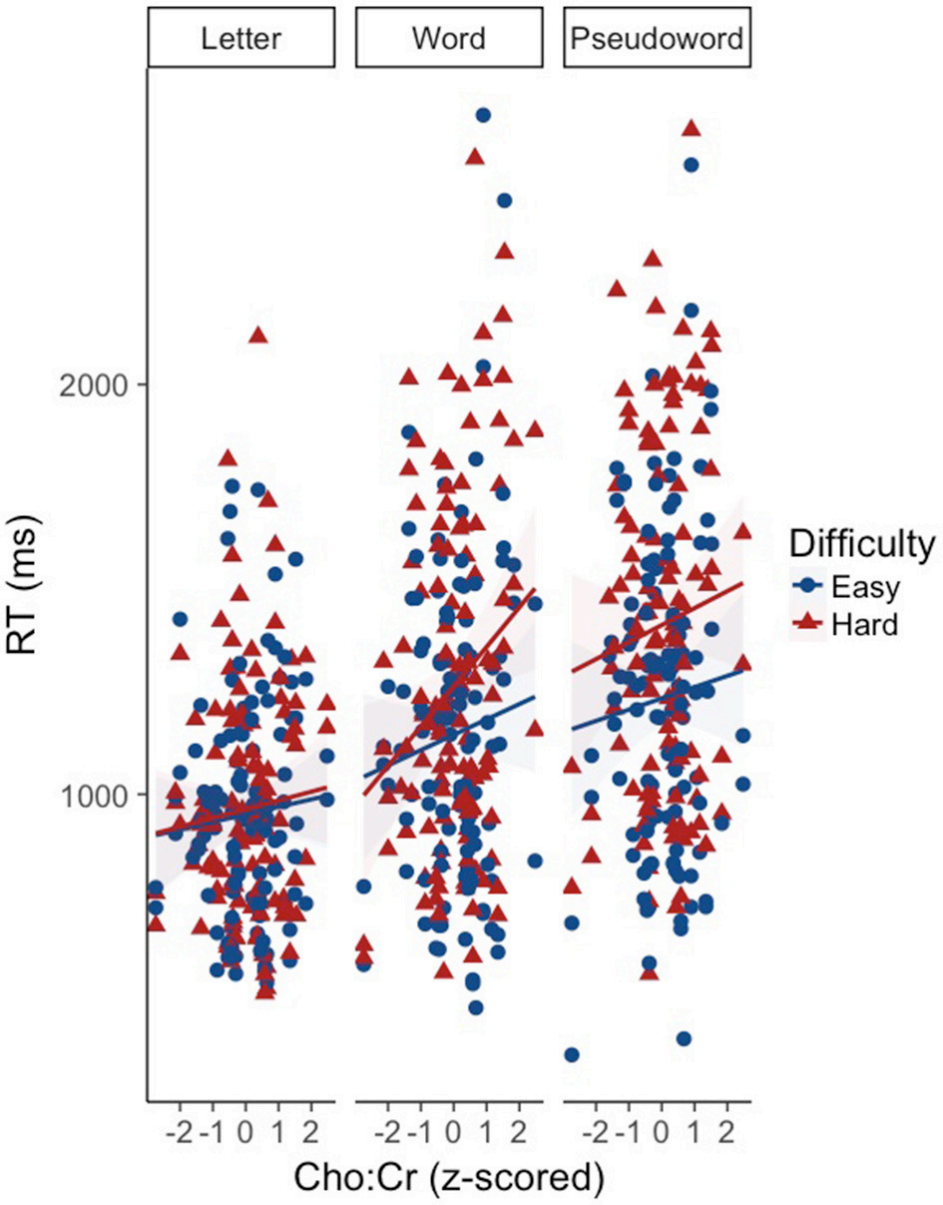
Effect of the interaction between Cho by stimulus condition by degree of difficulty on cross modal reaction time. Reaction time is reported in milliseconds (ms) on the y-axis. Cho:Cr concentration is reported on the x-axis. Stimulus condition is reported at the top of each panel. Difficulty is indicated by color and shape. The “hard” condition is in red triangles. The “easy” condition is in blue circles. The light blue and red areas reflect the standard error of the mean (SEM) for each difficulty condition.

There was an effect of GABA [*F*_(1, 65.82)_ = 10.39, *p* < 0.01] and NAA [*F*_(1, 66.30)_ = 8.62, *p* < 0.01] on CM-RT, where lower GABA and higher NAA concentrations predicted faster CM-RT (Figure 4). Moreover, there was a significant two-way interaction of stimulus condition by GABA [*F*_(2, 62.04)_ = 3.57, *p* < 0.05]. The two-way interaction of stimulus condition by GABA was driven by the word stimulus condition [*b* = 0.08, *SE* = 0.033, *t*_(61.40)_ = 2.53, *p* < 0.05] (Figure 5). These interactions again provide evidence, at least for the word condition, that lower GABA and higher NAA concentrations predict faster CM-RT. Additionally, there was a three-way interaction of stimulus condition by degree of difficulty by Cho [*F*_(3, 499.97)_ = 2.86, *p* < 0.05]. This interaction was significantly driven by the word [*b* = 0.04, *SE* = 0.019, *t*_(161.97)_ = 2.28, *p* < 0.05] condition, but not the pseudoword (*p* = 0.85) or letter (*p* = 0.93) stimulus conditions (Figure 6). Therefore, CM-RT in the hard word condition was faster for children with lower concentrations of Cho.

### Cross-modal matching mediates the effect of neurometabolite concentrations

Given that neurometabolite concentrations have previously been shown to have a negative association with reading ability (Pugh et al., 2014), we employed a mediation approach using SEM to test whether this relationship between neurometabolite concentration and reading ability was statistically mediated by cross-modal integration. Three latent variables were created. The latent variable (1) Reading Ability (RA) predicted Word Identification and Word Attack subtest scores (WJ-III: Woodcock et al., 2007), as well as Sight Word Efficiency and Phonemic Decoding Efficiency subtest scores (TOWRE: Torgesen et al., 1999). The latent variable (2) CM-RT predicted repetition 1 and repetition 2. Finally, the latent variable (3) neurometabolite predicted concentrations of Cho, Glu, GABA, and NAA. In addition, degree of difficulty was included as a categorical variable predicting CM-RT. The mediation model was a good fit [Maximum Likelihood *X*^2^ = 93.09, CFI = 0.937, RMSEA = 0.114 (90% CI: 0.083, 0.145), SRMR = 0.082; Robust (R.) Maximum Likelihood *X*^2^ = 91.426, R.CFI = 0.938, R.RMSEA = 0.113 (90% CI: 0.082, 0.144); and the scaling factor for the Yuan-Bentler correction was 1.018]. Specifically, the initial assumptions of mediation were met. There was a direct effect of increased neurometabolite concentration on slower CM-RT (*path a*: *b* = 0.61, *SE* = 0.26, *z* = 2.31, *p* < 0.05), as well as an effect of more difficult stimuli leading to slower CM-RT (*b* = 0.41, *SE* = 0.20, *z* = 1.98, *p* < 0.05). There was also a direct effect of faster CM-RT leading to better reading performance (*path b* = −0.47, *SE* = 0.09, *z* = 5.28, *p* < 0.001). When the indirect pathway was included (*b* = −0.29, *SE* = 0.14, *z* = 2.05, *p* = 0.040), the direct pathway from neurometabolite concentration to reading ability (*path c*: *b* = −0.76, *SE* = 0.32, *z* = 2.39, *p* = 0.017) was no longer significant (*path c-prime*: *p* = 0.23). The statistical mediation model confirms that individual differences in neurometabolite concentration influenced CM-RT, which in turn influenced reading ability.

#### Mediation analyses by stimulus condition

Mediation models were then used to examine if the relationship between neurometabolite concentration and reading ability was significantly influenced by CM-RT stimulus condition. Two latent variables were created. The latent variable (1) Reading Ability (RA) predicted Word Identification and Word Attack subtest scores (WJ-III: Woodcock et al., 2007), as well as Sight Word Efficiency and Phonemic Decoding Efficiency subtest scores (TOWRE: Torgesen et al., 1999). The latent variable (2) neurometabolite predicted concentrations of Cho, Glu, GABA, and NAA. Degree of difficulty was included as a categorical variable predicting CM-RT.

##### Letter condition

The mediation model for the letter condition was a very good fit [Maximum Likelihood *X*^2^ = 57.59, CFI = 0.97, RMSEA = 0.073 (90% CI: 0.040, 0.104), SRMR = 0.042, the R. Maximum Likelihood *X*^2^ = 56.88, R.CFI = 0.97, R.RMSEA = 0.073 (90% CI: 0.039, 0.104); and the scaling factor for the Yuan-Bentler correction was 1.013]. For the letter condition, there was a direct effect of faster CM-RT leading to better reading performance (*path b*: *b* = −0.34, *SE* = 0.08, *z* = 4.09, *p* < 0.001). However, there was no direct effect of neurometabolite concentration on CM-RT (*path a: p* = 0.30), nor was there an effect of stimulus difficulty (*p* = 0.85). Therefore, the mediating role of cross-modal matching was not driven by the letter condition.

##### Word condition

The mediation model for the word condition was a good fit [Maximum Likelihood *X*^2^ = 71.91, CFI = 0.96, RMSEA = 0.092 (90% CI: 0.063, 0.122), SRMR = 0.053, the R. Maximum Likelihood *X*^2^ = 70.63, R.CFI = 0.96, R.RMSEA = 0.091 (90% CI: 0.062, 0.121); and the scaling factor for the Yuan-Bentler correction was 1.02]. For the word condition, the initial assumptions of mediation were met. There was a direct effect of faster CM-RT for words leading to better reading performance (*path b*: *b* = −0.51, *SE* = 0.08, *z* = 6.66, *p* < 0.001). There was a direct effect of increased neurometabolite concentration on slower CM-RT (*path a*: *b* = 0.50, *SE* = 0.18, *z* = 2.80, *p* < 0.01), but only a trending effect of more difficulty stimuli leading to slower CM-RT for words (*p* = 0.086). When the indirect pathway was included (*path ab*: *b* = −0.26, *SE* = 0.097, *z* = 2.67, *p* = 0.008) the direct pathway from neurometabolite concentration to reading ability (*path c*: *b* = −0.76, *SE* = 0.32, *z* = 2.39, *p* = 0.017) was no longer significant (*path c-prime*: *p* = 0.53), indicating full statistical mediation.

##### Pseudoword condition

The mediation model for the pseudoword condition was a good fit [Maximum Likelihood *X*^2^ = 69.90, CFI = 0.96, RMSEA = 0.090 (90% CI: 0.060, 0.119), SRMR = 0.057, the R. Maximum Likelihood *X*^2^ = 69.60, R.CFI = 0.96, R.RMSEA = 0.90 (90% CI: 0.060, 0.119); and the scaling factor for the Yuan-Bentler correction was 1.004]. In the pseudoword condition, there was an effect of more difficult stimuli leading to slower CM-RT (*b* = 0.41, *SE* = 0.16, *z* = 2.22, *p* < 0.05). There was a direct effect of faster CM-RT leading to better reading performance (*path b*: *b* = −0.45, *SE* = 0.095, *z* = 4.78, *p* < 0.001). However, there was no direct effect of neurometabolite concentration on CM-RT (*path a: p* = 0.24). This suggests that the mediating role of cross-modal matching was not driven by the pseudoword condition.

#### Word mediation analyses by neurometabolite

SEM mediation models were then used to investigate if reading ability mediated by the effect of word CM-RT was predicted by specific neurometabolites (Glu and Cho), which have previously been linked to reading ability. Only one latent variable was included. The latent variable (1) Reading Ability (RA) predicted Word Identification and Word Attack subtest scores (WJ-III: Woodcock et al., 2007), as well as Sight Word Efficiency and Phonemic Decoding Efficiency subtest scores (TOWRE: Torgesen et al., 1999). As in the previous mediation model, degree of difficulty was included as a categorical variable predicting CM-RT.

##### Word CM-RT mediates the relationship between glu and reading ability

**Figure 7:**
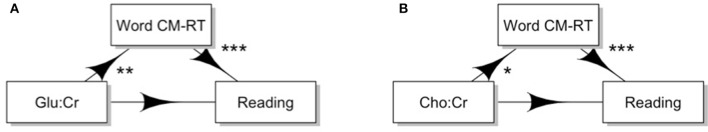
Cross-modal reaction time influences the effect of neurochemistry on reading ability. **(A)** Cross modal reaction time significantly mediates the relationship between Glu:Cr and reading ability. **(B)** Cross modal reaction time significantly mediates the relationship between Cho and reading ability. Significance: *p < 0.05, **p < 0.01, ***p < 0.001.

The mediation of the relationship between reading ability and Glu by word CM-RT was a good fit [Maximum Likelihood *X*^2^ = 27.86, CFI = 0.98, RMSEA = 0.098 (90% CI: 0.050, 0.146), SRMR = 0.033, the R. Maximum Likelihood *X*^2^ = 26.73, R.CFI = 0.98, R.RMSEA = 0.096 (90% CI: 0.047, 0.146); and the scaling factor for the Yuan-Bentler correction was 1.042]. There was a direct effect of faster CM-RT for words leading to better reading performance (*path b*: *b* = −0.51, *SE* = 0.08, *z* = 6.66, *p* < 0.001). There was a direct effect of increased Glu concentration on slower CM-RT (*path a*: *b* = 0.24, *SE* = 0.09, *z* = 2.80, *p* < 0.01), but only a trending effect of more difficult stimuli leading to slower CM-RT for words (*p* = 0.086). When the indirect pathway was included (*path ab*: *b* = −0.12, *SE* = 0.046, *z* = 2.65, *p* = 0.008) the direct pathway from Glu concentration to reading ability (*path c*: *b* = −0.22, *SE* = 0.072, *z* = 3.00, *p* = 0.003) was no longer significant (*path c-prime*: *b* = −0.10, *SE* = 0.07, *z* = 1.31, *p* = 0.19) (Figure 7A). This indicates that faster cross-modal CM-RT for words mediated the relationship between reading ability and Glu.

##### Word CM-RT mediates the relationship between cho and reading ability

Additionally, the mediation of the relationship between reading ability and Cho by word CM-RT was also a good fit [Maximum Likelihood *X*^2^ = 38.33, CFI = 0.97, RMSEA = 0.126 (90% CI: 0.083, 0.172), SRMR = 0.036, the R. Maximum Likelihood *X*^2^ = 37.51, R.CFI = 0.97, R.RMSEA = 0.125 (90% CI: 0.081, 0.172); and the scaling factor for the Yuan-Bentler correction was 1.022]. There was a direct effect of faster CM-RT for words leading to better reading performance (*path b*: *b* = −0.51, *SE* = 0.08, *z* = 6.66, *p* < 0.001). There was a direct effect of increased Cho concentration on slower CM-RT (*path a*: *b* = 0.19, *SE* = 0.09, *z* = 1.99, *p* < 0.05), but no effect of stimuli difficulty leading to slower CM-RT for words (*p* = 0.094). When the indirect pathway was included (*path ab*: *b* = −0.10, *SE* = 0.053, *z* = 1.84, *p* = 0.066) the direct pathway from Cho concentration to reading ability (*path c*: *b* = −0.16, *SE* = 0.084, *z* = 1.90, *p* = 0.06) was no longer marginally significant (*path c-prime*: *p* = 0.147) (Figure 7B). This indicates that faster CM-RT for words fully mediated the relationship between reading ability and Cho.

### Percent correct

Overall, children's performance was very high [full sample (*n* = 224): percent correct = 0.944, *SD* = 0.10 and subsample (*n* = 69): percent correct = 0.940, *SD* = 0.10]. Table 5 includes task percent correct by condition for both the full sample and the subsample.

**Table 5:** Cross modal task accuracy by stimulus condition.

**Condition**	**Full sample (*****n*** = **224)**			**Subsample (*****n*** = **69)**		
	**Accuracy**			**Accuracy**		
	***n***	***M***	***SD***	***n***	***M***	***SD***
Letter	224	0.97	0.06	69	0.96	0.06
Word	223	0.94	0.11	68	0.94	0.10
Pseudoword	221	0.92	0.13	67	0.92	0.13

*The total number includes only those participants who scored above chance. Sample mean (M) and standard deviation (SD) are reported. Accuracy is the percent correct*.

#### Percent correct (full sample)

A non-parametric *Friedman* test was employed to compare the total percent correct for the four measures (easy-repetition1, hard-repetition1, easy-repetition2, and hard-repetition2) of each stimulus condition in the full sample of participants. This included 224 children for the letter stimulus condition, 222 children for the word stimulus condition, and 221 children for the pseudoword stimulus condition. There was a significant difference between the four measures of each stimulus condition [*Friedman X*${}_{\left(11\right)}^{2}$ = 326.7, *p* < 0.0001]. We then investigated if differences were due to stimulus condition using an adjusted critical alpha (0.05/3 = 0.016). Significant differences were found on the four measures of the word [*X*${}_{\left(3\right)}^{2}$ = 141.38, *p* < 0.0001] and pseudoword [*X*${}_{\left(3\right)}^{2}$ = 100.3, *p* < 0.0001] stimulus conditions, but not the letter stimulus condition (*p* = 0.32). *Post-hoc* tests were carried out to determine if differences were due to repetition or degree of difficulty within each stimulus condition (0.05/12 = 0.00416). In the word stimulus condition, there was a significant difference between the hard and easy word stimuli on both the first repetition [*X*${}_{\left(1\right)}^{2}$ = 82.14, *p* < 0.0001] and the second repetition [*X*${}_{\left(1\right)}^{2}$ = 60.19, *p* < 0.0001]. There was no difference due to repetition of the easy words (*p* = 0.17) or the hard words (*p* = 0.58). The same was true for pseudowords, where there was a significant difference between the hard and easy pseudoword stimuli on both the first repetition [*X*${}_{\left(1\right)}^{2}$ = 55.19, *p* < 0.0001] and the second repetition [*X*${}_{\left(1\right)}^{2}$ = 42.82, *p* < 0.0001]. Again, there was no difference found due to repetition of the easy pseudowords (*p* = 0.30) or the hard pseudowords (*p* = 0.18). Thus, for both the word and pseudoword condition, differences in percent correct during cross-modal matching were driven by degree of difficulty.

#### Percent correct (subsample)

A non-parametric *Friedman* test was employed to compare the total percent correct for the four measures (easy-repetition1, hard-repetition1, easy-repetition2, and hard-repetition2) of each stimulus condition in the subsample of participants. This included 69 children for the letter stimulus condition, 68 children for the word stimulus condition, and 67 children for the pseudoword stimulus condition. There was a significant difference between the four measures of each stimulus condition [*Friedman X*${}_{\left(11\right)}^{2}$ = 72.04, *p* < 0.0001]. As in the prior full sample analysis, we investigated if differences were due to stimulus condition using an adjusted critical alpha (0.05/3 = 0.016). Significant differences were found on the four measures of the word [*X*${}_{\left(3\right)}^{2}$ = 20.55, *p* < 0.001] and pseudoword [*X*^2^_(3)_ = 34.17, *p* < 0.0001] stimulus conditions, but not the letter stimulus condition (*p* = 0.26). As in the prior full sample analysis, *post-hoc* tests were carried out to determine if differences were due to repetition or degree of difficulty within each stimulus condition (0.05/12 = 0.00416). In the word stimulus condition, there was a significant difference between the hard and easy word stimuli on both the first repetition [*X*${}_{\left(1\right)}^{2}$ = 18.67, *p* < 0.0001] and the second repetition [*X*${}_{\left(1\right)}^{2}$ = 7.54, *p* < 0.01]. There was no difference due to repetition of the easy words (*p* = 0.65) or the hard words (*p* = 0.32). The same was true for pseudowords, where there was a significant difference between the hard and easy pseudoword stimuli on both the first repetition [*X*${}_{\left(1\right)}^{2}$ = 18.69, *p* < 0.0001] and the second repetition [*X*${}_{\left(1\right)}^{2}$ = 14.24, *p* < 0.001]. Again, there was no difference found due to repetition of the easy pseudowords (*p* = 0.51) or the hard pseudowords (*p* = 0.86). Therefore, we see that even in our subsample, differences in percent correct during cross-modal matching were driven by degree of difficulty.

## Discussion

We asked first-grade children to complete a cross-modal matching task with three language stimulus conditions (letter, word, and pseudoword. Degree of difficulty (hard and easy stimuli) and stimulus repetition (first presentation and second presentation) were included as nested and crossed factors, respectively. As in previous multimodal studies, there was a high degree of individual variability on the cross-modal matching task. After accounting for individual differences in cross-modal performance, we found that CM-RT was the fastest for letters, followed by words, and slowest for pseudowords. CM-RT was faster in the easy words and pseudoword conditions; yet, no difference was found between the hard and easy letter condition. There was also an effect of repetition in the word and pseudoword stimulus conditions in the full sample, but the effect was not robust enough to be found in the subsample. Taken together, this indicates that by first grade prior knowledge of cross-modal matched stimulus pairs is already supported by information specific to real words (e.g., semantic information).

### Glu

The primary aim of our investigation was two-fold. First, we aimed to determine if neurochemical concentration predicted individual differences in readers' cross-modal integration. Second, given that neurometabolite concentrations have previously been shown to have a negative relationship with reading abilities (Bruno et al., 2013; Pugh et al., 2014), we aimed to provide insight into possible ways that cross-modal integration might influence the reationship between reading ability and neurometabolite concentration. Hancock et al. (2017) proposed that RD is the result of increased neural excitability, which leads to neural noise in cortical networks. Our colleagues suggested that a result of increased neural noise would be impairment in multisensory integration, due to robust multisensory encoding requiring that stimuli be spatially congruent and temporally synchronous (Meredith et al., 1987; Meredith and Stein, 1996; Kadunce et al., 1997). More specifically, Hancock et al. (2017) suggested that random and excessive variability in neuronal firing would lead to disruptions in neural synchronization and precise neural spike timing. This imprecision in synchronization would lead to impairments in multisensory integration. Our findings revealed that decreased Glu (our proximal measure of increased glutamatergic signaling and hyperexcitability) was associated with slower CM-RT, which was in turn associated with diminished reading performance. Interpreting our results in the framework of Hancock et al. (2017), this finding suggests that increased neural noise, due to increased glutamatergic signaling, corresponds to decreased multimodal integration, and thus lessened reading ability.

### GABA

The emergence of multisensory integration relies on GABA circuit maturation (Allman et al., 2008; Gogolla et al., 2014; Balz et al., 2016). Evidence from animal studies suggests that reorganization of the GABAergic system in early development is what leads to impaired or unimpaired multisensory integration (Gogolla et al., 2014). We found that a lower concentration of GABA in the visual cortex predicted faster cross-modal matching. This indicates that a lower GABA concentration allows children to quickly integrate and match auditory and visual stimuli, resulting in faster CM-RT. In MRS studies, increased GABA is often found to indicate increased performance on speeded tasks (Boy et al., 2010). However, this is typically achieved through increased motor inhibition, which results in slower reaction time (Stagg et al., 2011a,b). Our cross-modal findings are in fact consistent with recent evidence from Nakai and Okanoya (2016), who reported that lower GABA predicted increased reading fluency in the left (but not right) inferior frontal gyrus (IFG). In their study, reading fluency was assessed by having adults quickly write down nouns belonging to a category (e.g., fruit).

GABA's role in metabolite energetics is often tightly coupled with Glutamate (Patel et al., 2005; Ramadan et al., 2013). Sensory stimuli neural encoding time windows are tightly linked to neural excitability, and this excitation triggers a shadowing period of inhibition. It is during this period of inhibition that sensory input is integrated prior to the next excitatory neuronal spike. This largely explains why multimodal integration and coordination across cortical regions are particularly sensitive to the loss of spike timing precision, due to their occurrence over a restricted time window (Senkowski et al., 2007a,b). Greater GABA concentration leads to more selective cortical tuning, resulting in greater perceptual acuity (Kolasinski et al., 2017). Likewise, GABA correlates with unisensory visual perception and is highly predictive of individual performance (Edden et al., 2009). Therefore, our result of decreased GABA leading to faster CM-RT likely allows children to more quickly integrate already learned cross-modal stimulus pairs, but that this may not be possible for readers that require more perceptual acuity or time to encode and differentiate sensory information. In other words, less proficient readers are more likely to require increased GABA to support cross-modal matching performance, while our skilled first-grader readers are proficient enough that increased GABA is not necessary for perceptual acuity and likely hinder their reaction time.

GABA negatively correlates with the functional Magnetic Resonance Imaging (fMRI) blood-oxygen-level dependent (BOLD) signal (Northoff et al., 2007; Donahue et al., 2010). Cross-modal deactivation has been reported in fMRI, but deactivation was not present during paired stimulus presentations (Laurienti et al., 2002). This is supported by evidence from *in vivo* whole-cell recordings, where Iurilli et al. (2012) found cross-modal influence of the auditory cortex on inhibitory GABAergic circuits in the primary visual cortex. Moreover, GABA contributes to the generation of gamma band oscillations (e.g., Traub et al., 2003; Bartos et al., 2007). During maturation, GABA signaling is a powerful regulatory mechanism of parvalbumin (PV) cell innervation patterns (Chattopadhyaya et al., 2007; Gogolla et al., 2014). Inhibition of PV interneurons suppressed gamma oscillations, while excitation of PV interneurons generates emergent gamma-frequency rhythmicity (Sohal et al., 2009). The rate of gamma oscillations is also highly predictive of multisensory integration (Kaiser and Lutzenberger, 2005; Hipp et al., 2011) and is abnormally fast in RD (Lehongre et al., 2011, 2013). Intriguingly, gamma-frequency modulation of excitatory input was found to enhance signal transmission through output to PV interneurons and reduce neural circuit noise (Sohal et al., 2009). This mechanism likely accounts for the recent finding that GABA mediates the relationship between gamma band oscillation and audiovisual integration (Balz et al., 2016).

Terhune et al. (2014) reported that GABA concentrations in the motor and visual cortex were independent of each another. In our study, the MRS spectra was collected from a voxel on the midline of the occipital cortex. Thus, in considering the relationship between lower GABA and cross-modal matching speed, it is also critical to consider the GABA plays a nuanced role that is dependent upon brain location. The single voxel spectroscopy location is a limitation of the current study; indeed, current studies are underway to examine more holistically the role that neurochemistry plays in reading ability (Pugh and Hoeft, 2017).

### Cho

Consistent with previous research findings that Cho negatively predicted reading ability (e.g., Bruno et al., 2013; Pugh et al., 2014 from the NIH MRI Study of Normal Brain Development: http://pediatricmri.nih.gov, release 5), we found that Cho predicted cross-modal word matching speed; specifically, lower Cho predicted faster cross-modal matching for hard words. Moreover, decreased Cho was associated with faster CM-RT in the word stimulus condition, which was in turn associated with better reading performance. The Cho signal measured in proton MRS corresponds largely to glycerophosphocholine (GPCho), phosphocholine (PCho), and free choline (Miller, 1991). These compounds are products and building blocks for membrane metabolism, and have been proposed to function in the osmotic regulation of cell volume, as well as support cell proliferation and differentiation (Brenner et al., 1993; Jackowski, 1994; Kwon et al., 1995). Proton MRS measures of Cho have been associated with myelination (Laule et al., 2007), neurodegeneration or inflammation due to membrane/phospholipid turnover (Roser et al., 1995), as well as cellular density (Miller et al., 1996).

Bruno et al. (2013) suggested that decreased Cho is linked to phonological processing. This conclusion was based on adult neurochemical concentrations of Cho, accounting for a additional variance in phonological decoding of pseudowords, beyond word reading. The authors “tentatively [suggest that this] indicates some specificity for the negative relationship between Cho and phonological decoding.” However, they also reported that word and pseudoword decoding showed the same relationship with Cho, but that the relationship was more robust for pseudoword decoding. Moreover, they report a moderate degree of overlap in the variance accounted for by word and pseudoword decoding. Based on these findings Bruno et al. (2013) suggested some alternative explanations for the results of their study. One such explanation was that difficulty of the linguistic stimuli may be what drives these differences. In keeping with this alternative explanation, our work similarly suggests that difficulty of the linguistic stimuli likely plays a role in the relationship to Cho. Thus, we hesitate to make any claims regarding specific linguistic constructs, but instead suggest that degree of difficulty of the linguistic stimuli likely drives differences in Cho concentration between letter, word, and pseudoword CM-RT.

### NAA

NAA is a marker of neuronal viability and is considered to be a neurochemical correlate of neuron-oligodendrocyte (axon-myelin) integrity (Moffett et al., 2007; Paslakis et al., 2014; Xu et al., 2016). NAA has been reported to correspond to measures of diffusion weighted imaging (Caprihan et al., 2015). Here, higher concentrations of NAA predicted faster cross-modal matching. Individual developmental differences in cross-modal brain activation has been found to correspond to connectivity in the arcuate fasciculus (Gullick and Booth, 2014). Our interpretation of these findings is that a more intact white matter reading network likely corresponds to higher NAA. There are now several studies that have linked measures of the integrity of the left arcuate fasciculus to reading skill (Yeatman et al., 2011) and longitudinal reading change (Gullick and Booth, 2015). Future work examining individual differences in the white matter reading network and longitudinal changes in reading development may benefit from investigating the corresponding role played by NAA.

## Conclusion

In summary, this work provides supporting evidence of the *Neural Noise Hypothesis of Developmental Dyslexia* (Hancock et al., 2017), and allows us to better understand the role of neurochemistry in reading disability. Specifically, this work shows that Glu and Cho concentrations influence cross-modal matching, which in turn effects reading ability. This study is the first to demonstrate a direct relationship between individual differences in cross-modal matching and emergent readers' GABA and NAA neurochemical concentrations. Further, this work links behavioral studies of multisensory phonological and orthographic integration and reading performance with pediatric Magnetic Resonance Spectroscopy (MRS) studies.

## Ethics statement

This study was carried out in accordance with the recommendations of Yale University's Human Research Protection Program. The protocol was approved by the Yale University's Human Research Protection Program. All participants' parents provided written informed consent, while children gave written assent, in accordance with the Declaration of Helsinki.

## Author contributions

SD, SF, PM, GM, DR, RF, and KP designed and performed the research. SD, PM, and GM analyzed the data. GM, DR, and RF contributed unpublished reagents and, analytic tools. SD wrote the manuscript with assistance from SF, FH, LC, PM, GM, DR, RF, and KP.

### Conflict of interest statement

GM is a consultant for Sumitomo Dainippon Pharma Co. Ltd. and UCB Pharma SA, and serves on the Scientific Advisory Board of Elucidata Inc.

The remaining authors declare that the research was conducted in the absence of any commercial or financial relationships that could be construed as a potential conflict of interest.
