# Supplementary material for: Neurochemistry Predicts Convergence of Written and Spoken Language: A Proton Magnetic Resonance Spectroscopy Study of Cross-Modal Language Integration
Source: Front Psychol. 2018 Sep 4;9:1507. doi: 10.3389/fpsyg.2018.01507 (PMC6131664; doi:10.3389/fpsyg.2018.01507)
Supplement: Supplementary file 1 [file Table_1.DOCX]

Appendix A

Letter Stimuli: Included all 26 characters of the English alphabet.

Word Stimuli: *BAG, BAR, BEG, BIG, BUG, BUS, CAB, CAT, COP, COW, CUP, CUT, DIG, DOG, HIP, HIT, FAN, FAT, FUN, FUR, JAM, JAW, JOB, JOY, LID, LIP, LOT, LOW, MAD, MOM, MOP, MUD, NET, NUT, PAL, PAY, POT, PUT, RAG, RAT, RUG, RUN, SAD, SAY, SET, SIR, SIT, SIX, TAN, TEN, TOP, TOY, WEB, WET, WIG, WIN*

Pseudoword Stimuli: *BAL, BAF, BIM, BIP, CAG, CUG, DEM, DER, DOP, DOY, DUP, DUT, FAP, FAS, FEG, FET, FID, FOD, FUB, FUG, FUM, FUT, GAD, GAN, GAR, GAT, HAB, HEB, JAD, LAR, LAT, LEF, JAT, LEB, MIM, MIP, MOG, MOT, NEN, NIN, REN, RIN, SEF, SEM, SIG, SIM, TID, TIG, TUD, TUP, VOG, VOT, WID, WIS, ZOP, ZOT*
